# Depression, Anxiety, and Stress Symptoms Among Students in Croatia During the COVID-19 Pandemic: A Systematic Review

**DOI:** 10.3390/jcm13206240

**Published:** 2024-10-19

**Authors:** Stipe Vidović, Slavica Kotromanović, Zenon Pogorelić

**Affiliations:** 1 Faculty of Medicine, Josip Juraj Strossmayer University of Osijek, 31 000 Osijek, Croatia; 2 Department of Neurosurgery, University Hospital of Split, 21 000 Split, Croatia; 3 Department of Surgery, School of Medicine, University of Split, 21 000 Split, Croatia; 4 Department of Pediatric Surgery, University Hospital of Split, 21 000 Split, Croatia

**Keywords:** depression, anxiety, stress, mental health, mental disorder, COVID-19, student, Croatia

## Abstract

**Background:** The COVID-19 pandemic has led to an increased fear of infection, social isolation, financial concerns, and feelings of loneliness and uncertainty. Studies reveal that the pandemic has had a significant negative impact on mental health. This systematic review aimed to investigate the prevalence of depression, anxiety, and stress symptoms among students in Croatia during the COVID-19 pandemic. **Methods:** A systematic review was conducted following PRISMA guidelines. A literature search was performed on August 5, 2024, using the electronic databases Scopus, PubMed, Web of Science, and PsycINFO. The search utilized the Boolean logical operator expression (‘depression’ AND ‘anxiety’ AND ‘stress’ AND ‘COVID-19’ AND ‘Croatia’ AND ‘student’). **Results:** Out of one hundred and fifty-four identified studies, five met the inclusion criteria and were included in the review. The studies used the Generalized Anxiety Disorder 7-item scale (GAD-7), the Patient Health Questionnaire-9 (PHQ-9), and the Depression, Anxiety, and Stress Scale-21 (DASS-21) to assess mental health. The results indicate a high prevalence of symptoms of depression, anxiety, and stress among students in Croatia during the pandemic. **Conclusion:** A high prevalence of negative affective emotions was observed among students in Croatia during the pandemic. This finding underscores the importance of developing effective strategies for the early identification and management of mental disorders among students, irrespective of the pandemic’s conclusion.

## 1. Introduction

Mental disorders represent a significant global public health challenge in the modern era [1]. According to the World Health Organization, one in eight individuals worldwide is affected by a mental disorder [2]. Numerous preventive programs and interventions have been implemented with the aim of reducing the increasing prevalence of mental disorders and mitigating their negative consequences. Nevertheless, despite these efforts, a global rise in the prevalence of mental disorders has been documented since 1990 [3,4,5,6].

Studies indicate that many mental disorders have their highest incidence during adolescence [7,8]. One of the risk factors contributing to the high levels of negative emotions and increased vulnerability to the development of mental disorders during adolescence and early adulthood is participation in tertiary education [1,9]. Meta-analyses and systematic reviews describe that students exhibit higher levels of depression and anxiety compared to their peers who do not attend tertiary education [10,11]. The high prevalence of affective disorders among students is attributed to various factors that students encounter during their education. Academic pressures, along with changes in sleep, dietary, and physical activity patterns, often exacerbate negative emotions within the student population [12,13,14,15]. Additionally, adjusting to new social environments frequently leads to feelings of isolation and homesickness, particularly among students who have relocated far from home [16]. Moreover, the increased use of digital and social media often intensifies feelings of anxiety and depression among students due to social comparisons and reduced face-to-face interactions [17,18]. Furthermore, the lack of adequate support systems within educational institutions and limited access to mental health services leave many students without the necessary assistance, allowing symptoms of mental disorders to escalate [19,20].

The COVID-19 pandemic was characterized by an increased fear of infection, feelings of loneliness and uncertainty, social isolation, and financial concerns [21,22]. Systematic reviews and meta-analyses reveal that the COVID-19 pandemic had a significant negative impact on people’s mental health [1,11,23,24,25]. A more pronounced effect on mental health was observed among young people, women, and individuals with pre-existing mental disorders [24]. Moreover, studies suggest that the pandemic significantly contributed to the increased prevalence of depression, anxiety, and stress among students on a global scale [11,15,25]. A meta-analysis by Ahmed et al. (2023) indicated that levels of depression and anxiety among students in Europe during the pandemic were higher compared to pre-pandemic levels [26]. When comparing the prevalence of mental disorders during the pandemic between the student population and the general population worldwide, a higher incidence of mental disorders was observed among students [27]. Additionally, Daniali et al. noted in their meta-analysis and systematic review that students in Europe experienced higher levels of depression, anxiety, and stress during the pandemic compared to the general population [11]. Wang et al. conducted a systematic review and meta-analysis (2020) examining the prevalence of depression, anxiety, and stress among students globally during the COVID-19 pandemic. Their analysis revealed that 37% of students exhibited symptoms of depression, 29% experienced anxiety, and 23% reported stress symptoms [26]. Furthermore, Zhang et al., in their systematic review and meta-analysis (2022), found that among students in Eastern Europe, 32% reported symptoms of depression, while 31% exhibited symptoms of anxiety [25].

The COVID-19 pandemic was officially declared over on May 5, 2023, and the aforementioned studies were conducted prior to this, limiting the scope of the analysis on student mental health during the pandemic. With the pandemic now concluded, this systematic review aims to examine the prevalence of depression, anxiety, and stress symptoms in the student population in Croatia throughout the COVID-19 pandemic.

## 2. Methods

### 2.1. Literature Search Strategy

A systematic literature review was conducted in accordance with the Preferred Reporting Items for Systematic Reviews and Meta-Analyses (PRISMA) guidelines [28]. The search was performed by reviewer S.V. on 5 August 2024, using four electronic databases: Scopus, PubMed, Web of Science, and PsycINFO. The search within these databases was conducted using a Boolean logical operator expression, (‘depression’ AND ‘anxiety’ AND ‘stress’ AND ‘COVID-19’ AND ‘Croatia’ AND ‘student’), with restrictions to studies published in English and Croatian, focused on the territory of Croatia, and published within the last 10 years. The inclusion and exclusion criteria for the studies in the systematic review are listed in Table 1.

### 2.2. Data Extraction

For each study included in the systematic review, the following data were extracted: the first author of the article, the year of publication, study design, the total number of participants, gender distribution, study period, and the instrument used to assess the prevalence of depression, anxiety, and stress symptoms. Data extraction was conducted by two reviewers (S.V. and Z.P.).

### 2.3. Outcomes of the Study

The systematic review assessed the prevalence of depression, anxiety, and stress among students in Croatia during the COVID-19 pandemic using a reliable instrument for measuring symptoms of negative affective emotions. Additionally, the review provided insights into the severity levels of these symptoms within the student population.

### 2.4. Assessment of the Methodological Quality of Studies

Two independent reviewers (S.V. and Z.P.) assessed the methodological quality and potential sources of bias in the included studies using the Joanna Briggs Institute (JBI) Critical Appraisal Checklist for Studies Reporting Prevalence Data [29].

The Joanna Briggs Institute (JBI) Critical Appraisal Checklist for Studies Reporting Prevalence Data is a comprehensive tool designed to assess the methodological quality of studies reporting prevalence data. This checklist is widely used in systematic reviews and meta-analyses to evaluate the reliability and validity of prevalence studies [29].

This tool comprises nine questions, which are as follows: ‘Was the sample frame appropriate to address the target population?’; ‘Were study participants sampled in an appropriate way?’; ‘Was the sample size adequate?’; ‘Were the study subjects and the setting described in detail?’; ‘Was the data analysis conducted with sufficient coverage of the identified sample?’; ‘Were valid methods used for the identification of the condition?’; ‘Was the condition measured in a standard, reliable way for all participants?’; ‘Was appropriate statistical analysis conducted?’; and ‘Was the response rate adequate, and if not, was the low response rate managed appropriately?’ [29].

Reviewers (S.V. and Z.P.) responded to these questions with either ‘Yes’, ‘No’, ‘Unclear’, or ‘Not applicable’. Disagreements between the reviewers at various stages of the review were resolved through discussion. A ‘Yes’ response contributed one point, while other responses did not contribute points. The total score, ranging from 0 to 9, was the sum of all ‘Yes’ responses. The overall quality assessment score was calculated by dividing the total score by the maximum possible score, expressed as a percentage. Methodological quality was ranked as low (less than 33%), medium (33–66%), or high (over 66%).

Upon assessing the methodological quality of the studies, three were found to be of high quality, while two were rated as medium quality according to the total quality assessment score. The results of the evaluation of the included studies, conducted using the JBI Critical Appraisal Checklist for Studies Reporting Prevalence Data, are presented in Table 2.

### 2.5. Statistical Analysis

The results for nominal variables were presented as absolute and relative frequencies (percentages). The collected data were processed using the Microsoft Office 2021 software package. Data were analyzed using Microsoft Excel for Windows version 16.0 (Microsoft Corporation, Redmond, WA, USA).

## 3. Results

### 3.1. Study Characteristics

**Figure 1:**
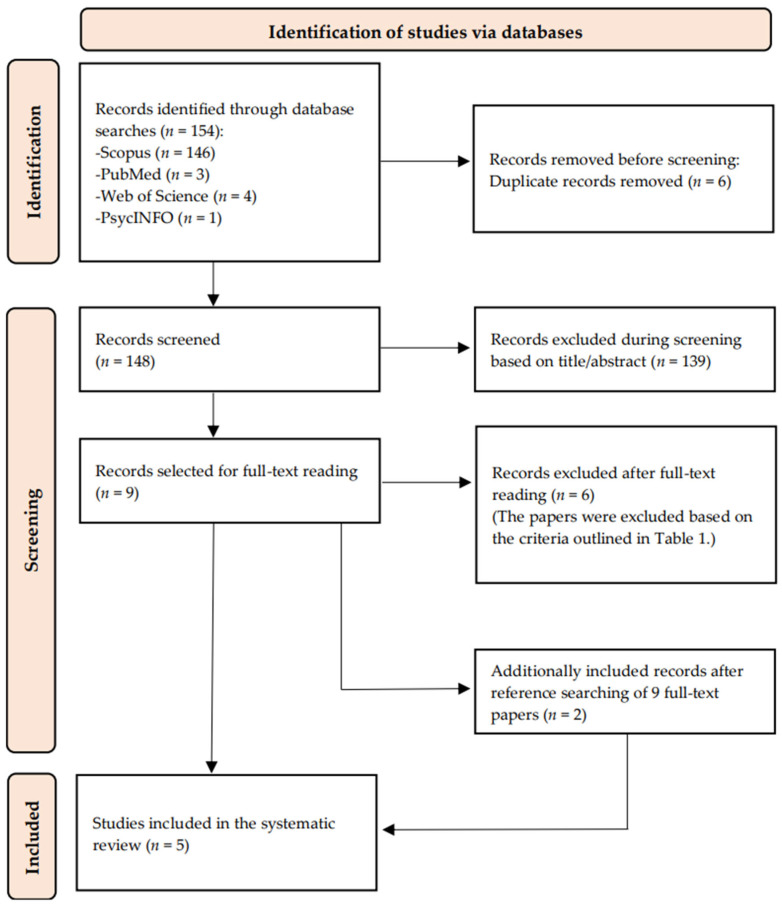
PRISMA flow diagram of the literature search (Table 1).

The database search retrieved one hundred and fifty-four records, six of which were identified as duplicates and removed before the screening phase. In the screening phase, based on titles and abstracts, 139 records were excluded. After screening, nine papers were read in full, and six were excluded based on inclusion and exclusion criteria (Table 1). Additionally, two records were included after manually searching the reference lists of selected records and the reference lists of recently published meta-analyses and systematic reviews that addressed the topic of students’ mental health during the COVID-19 pandemic. Ultimately, five studies were included in the systematic review. The PRISMA flow diagram of the literature search is presented in Figure 1.

The studies included in the systematic review were cross-sectional. For mental health assessment, they utilized the 9-item Patient Health Questionnaire (PHQ-9), Generalized Anxiety Disorder 7-item (GAD-7), or the Depression, Anxiety, and Stress Scale-21 (DASS-21). These tools are widely employed in both clinical and research settings due to their reliability and ease of administration [35,36,37].

The PHQ-9 is a well-established instrument used to assess the presence and severity of depressive symptoms based on the diagnostic criteria for major depressive disorder (MDD) from the Diagnostic and Statistical Manual of Mental Disorders, fourth edition (DSM-IV). It consists of nine items, each addressing a specific symptom of depression, such as diminished interest in activities, changes in appetite or sleep, fatigue, and thoughts of self-harm. Respondents are asked to rate how often they have experienced these symptoms over the past two weeks on a four-point Likert scale ranging from zero (“Not at all”) to three (“Nearly every day”). The total PHQ-9 score is then calculated, based on which participants are categorized as either exhibiting depressive symptoms or not, with higher total scores indicating more severe depressive symptoms [35].

The GAD-7 is a similarly structured self-report questionnaire developed to assess the presence and severity of generalized anxiety disorder (GAD). Comprising seven items, the GAD-7 captures core symptoms of GAD, such as excessive worry, difficulty controlling anxiety, restlessness, and irritability. Like the PHQ-9, respondents rate the frequency of symptoms over the past two weeks on a four-point Likert scale. The total score indicates the severity of anxiety, and participants are categorized as either having or not having anxiety symptoms [36].

The DASS-21 is a multidimensional measure designed to assess symptoms across three domains: depression, anxiety, and stress. This instrument is a shortened version of the original 42-item DASS and contains 21 items, with 7 items allocated to each domain. The depression subscale assesses symptoms such as hopelessness, lack of interest, and low mood; the anxiety subscale measures autonomic arousal, situational anxiety, and subjective feelings of fear; and the stress subscale captures persistent tension, nervousness, and difficulty relaxing. Respondents rate each item based on their experience over the past week, using a four-point Likert scale ranging from zero (“Did not apply to me at all”) to three (“Applied to me very much or most of the time”). Scores for each domain are summed to provide a total score for each construct, based on which participants are categorized as either exhibiting or not exhibiting symptoms of depression, anxiety, and stress. Additionally, higher scores indicate more severe symptoms [37].

The main characteristics of the studies included in the systematic review are shown in Table 3.

### 3.2. Summary of the Included Studies

Živić-Bećirević et al. conducted a cross-sectional study in May 2020, during the first wave of the COVID-19 pandemic, examining symptoms of depression among students at the University of Rijeka. The DASS-21 was used for this assessment. The study involved 923 students aged 19 to 28 years. A significant prevalence of depression was observed, with 51.6% of respondents exhibiting depressive symptoms. Of these, 11.5% of respondents expressed mild symptoms of depression, 20.5% moderate, 8.1% severe, and 11.5% extremely severe. The study also explored suicidal ideations and attitudes toward seeking professional psychological help among students in Croatia during the pandemic. Notably, 12.7% of students reported having suicidal ideations, yet only 4.6% pursued professional psychological support [30].

Talapko et al. examined the mental health of students from the Faculty of Dental Medicine and Health Osijek at the University of Osijek during the third wave of the COVID-19 pandemic (from 26 November 2020 to 31 January 2021). The study included students from the Integrated Undergraduate and Graduate University Study Programme of Dental Medicine and the Undergraduate and Graduate University Study Programme of Nursing. The DASS-21 was utilized. The study included 823 students, revealing high levels of symptoms of depression (50.8%), anxiety (50.9%), and stress (49.9%). Of these, 11.3% had mild, 18.3% moderate, 10% severe, and 11.2% extremely severe symptoms of depression. Furthermore, 13.9% had mild, 8.9% moderate, 7.5% severe, and 20.7% extremely severe symptoms of anxiety. Mild stress symptoms were reported by 11.9% of respondents, moderate by 12.5%, severe by 14.6%, and extremely severe by 10.9% [31].

In January 2021, during the third wave of the pandemic, Romić et al. investigated depression and anxiety levels among medical students at the University of Zagreb using the GAD-7 and PHQ-9. The study included 280 students, of whom 65.2% exhibited symptoms of depression, and 75.3% showed symptoms of anxiety. Additionally, 33.3% of respondents expressed mild, 22.8% moderate, 6% moderately severe, and 3% severe symptoms of depression. Mild anxiety symptoms were expressed by 44.8% of respondents, moderate by 23.3%, and severe by 9.2% [32].

From 1 September to 20 September 2021, during the fourth wave of the pandemic, Šimleša et al. conducted a cross-sectional study on medical students in Croatia, examining the presence of symptoms of depression, anxiety, and stress. The study involved 206 medical students from the Faculty of Medicine at the University of Zagreb. To assess symptoms of depression, anxiety, and stress, they used the DASS-21. The study found that 25.7%, 26.7%, and 15% of students exhibited symptoms of depression, anxiety, and stress, respectively [33].

During the COVID-19 pandemic in March 2023, Milić et al. conducted a cross-sectional study examining levels of anxiety and depression among students in Croatia. All students were from health science disciplines, including nursing, dental hygiene, physiotherapy, medical laboratory diagnostics, midwifery, radiological technology, occupational therapy, and sanitary engineering. Anxiety was assessed using the GAD-7, and depression using the PHQ-9. A total of 2137 students participated in the study, with 76.5% of respondents exhibiting symptoms of anxiety, and 51.2% showing symptoms of depression. Of those, 36.8% expressed mild, 23.9% moderate, and 15.8% severe levels of anxiety symptoms. Additionally, 20% expressed mild, 11.5% moderate, 8% moderately severe, and 1.8% severe symptoms of depression [34].

A summary of the prevalence of depression, anxiety, and stress symptoms among students in Croatia during the pandemic is presented in Table 4.

## 4. Discussion

This study is the first systematic review to examine the mental health of the student population in Croatia during the COVID-19 pandemic. The results indicate a high prevalence of depression, anxiety, and stress symptoms among students.

Wang et al. conducted a systematic review and meta-analysis in 2020 that investigated the prevalence of depression, anxiety, and stress among students globally during the pandemic [38]. The study included 28 studies with a total of 436,799 participants, utilizing the DASS-21, GAD-7, and PHQ-9. The meta-analysis revealed that 37% (95% CI, 32–42%) of students reported symptoms of depression, 29% (95% CI, 19–25%) reported symptoms of anxiety, and 23% (95% CI, 8–39%) reported symptoms of stress. Studies by Živić-Bećirević et al., Talapko et al., Romić et al., and Milić et al. indicate that students in Croatia experienced higher levels of depression, anxiety, and stress compared to students globally [30,31,32,34]. In contrast, the study by Šimleša and Margetić found that Croatian students reported similar levels of these negative affective emotions as those reported globally during the pandemic [33,38].

Furthermore, Zhang et al. conducted a systematic review and meta-analysis in 2022 on students in Eastern Europe, also examining the prevalence of depression, anxiety, and stress during the pandemic [39]. This analysis included 21 studies with a total of 25 246 participants, utilizing various assessment instruments, including the DASS-21, GAD-7, PHQ-9, Hospital Anxiety and Depression Scale (HADS), Beck Depression Inventory (BDI), and Brief Symptom Inventory (BSI). The meta-analysis revealed that 32% (95% CI: 16–50%) of students in Eastern Europe reported symptoms of depression, while 31% (95% CI: 15–31%) reported symptoms of anxiety. In comparison, students in Croatia reported similar [30] or slightly higher levels of depression and anxiety than their counterparts in Eastern Europe [31,32,33,34].

The systematic review did not include studies that examined the mental health of students at the University of Split, as no research was found using reliable and validated questionnaires/instruments for assessing negative affective emotions, such as depression, anxiety, and stress. However, a cross-sectional study by Bećica et al. (2022), involving 466 students from the University of Split, posed two questions in which students self-assessed the frequency of feeling anxious and stressed during the pandemic. Respondents answered using one of the provided options: never; rarely; sometimes; or often. The study results indicate that 34.5% of students often felt anxious, and 34.3% reported often being under stress during the pandemic [40]. Furthermore, a cross-sectional study by Žuljević et al. (2021) examined the impact of the first COVID-19 lockdown on burnout among medical students in Split, Croatia, using the Copenhagen Burnout Inventory and Oldenburg Burnout Inventory [41]. Data were collected via surveys at two points: before the COVID-19 outbreak in Croatia (December 2019 and January 2020) and during the national lockdown, when epidemiological restrictive measures were implemented, including a switch to complete e-learning (1–20 June 2020). It was observed that students exhibited moderate levels of personal burnout, exhaustion, and disengagement. However, the study did not find a significant difference in burnout levels among students before and after the COVID-19 lockdown measures. This could be attributed to the short follow-up period of the study, suggesting that the negative effects of the pandemic on students’ mental health may not have reached their full potential at that time.

The pandemic lasted several years, with distinct phases/waves. Longitudinal studies described varying dynamics in the prevalence of mental illnesses among adolescents [42]. A systematic review and meta-analysis of longitudinal studies examining students’ mental health during the pandemic by Lee et al. (2023) found an increasing prevalence of moderate to severe symptoms of depression and anxiety as the pandemic progressed [43]. In our systematic review, it was observed that the study by Šimleša et al. had the smallest sample size and the lowest prevalence of symptoms of depression, anxiety, and stress [33]. This may be explained by the fact that sampling was conducted in September 2021, during the summer holidays, when students were not additionally burdened by academic obligations. Furthermore, Romić et al., in January 2021, like Šimleša et al., examined the mental health of students at the Zagreb School of Medicine but found a higher prevalence of depression, anxiety, and stress symptoms [32]. This could be attributed to the fact that, during this period, students were living in the Zagreb area, which was affected by several earthquakes, further intensifying the negative emotions already exacerbated by the pandemic. Additionally, the observed differences might be explained by the fact that, during the Šimleša et al. study (the fourth wave of the pandemic), national epidemiological measures were less strict compared to the periods during which the studies by Živičić-B et al. (first wave), Romić et al. (third wave), and Talapko et al. (third wave) were conducted [30,31,32]. A significant observation was made by Milić et al., indicating a high prevalence of depression and anxiety among students toward the end of the pandemic [34]. This finding underscores the need for further studies to assess students’ mental health and associated risk factors, with the aim of potentially describing the long-term effects of the pandemic on students’ mental health.

In Croatia, Galić and colleagues (2020) conducted a cross-sectional study examining the mental health of the general population during the pandemic. The study included 1244 participants, of whom 14% reported symptoms of depression and 31% reported symptoms of anxiety, suggesting that the general population in Croatia experienced lower levels of depression and anxiety during the pandemic compared to the student population [44]. This is consistent with the findings of a systematic review and meta-analysis by Daniali et al. (2023), which found that, on a global scale, students exhibited higher levels of depression, anxiety, and stress during the pandemic than the general population [11].

Overall, the results of the studies included in this systematic review indicate a high prevalence of negative affective states among students, which may be attributed to several interrelated factors. The academic demands and pressures typical of student life, such as stringent deadlines and high-stakes exams, can potentially intensify negative affective emotions [45]. Additionally, many students undergo a transition phase where they adjust to new social environments and may experience feelings of isolation or homesickness, particularly those who move away from home [46]. The increased use of digital and social media platforms among students can also contribute to anxiety and depression, as these platforms may lead to comparisons with peers and reduced face-to-face interactions [47]. Moreover, inadequate support systems at educational institutions and limited access to mental health services can leave many students without necessary psychological help, allowing symptoms of mental disorders to escalate unchecked [20]. Additionally, the COVID-19 pandemic may have exacerbated these issues by introducing several interconnected factors. These include increased academic uncertainties due to remote learning and changes in assessment methods, heightened social isolation from physical distancing measures and reduced in-person interactions, and financial concerns related to job losses or economic instability affecting students and their families [48,49,50]. Disruptions to daily routines, including altered sleep patterns and reduced physical activity, as well as the overall uncertainty and fear surrounding the pandemic, may have further aggravated these mental health issues [51,52,53,54]. Furthermore, the observed high levels of negative emotions among students could be explained by the fact that the participants in the studies conducted by Talapko et al., Romić et al., Šimleša et al., and Milić et al. were students of medicine or other health-related fields [31,32,33,34]. Students in these disciplines are typically more exposed to illness and death than their peers in other fields of study, and during the pandemic, they faced an intensified and dramatic rise in infection and mortality rates, which may have contributed to elevated levels of depression, anxiety, and stress among these students [55,56].

In addition to the high prevalence of depression, anxiety, and stress, a high prevalence of suicidal ideation and a low prevalence of seeking professional psychological support were observed among students at the University of Rijeka, Croatia [30]. To our knowledge, only one other study has examined attitudes toward seeking professional psychological help among students in Croatia during the pandemic. This study, conducted by Bečica et al. (2022), involved 466 students from the University of Split, Croatia, of whom only 11.8% sought such help. Furthermore, the study highlighted that 51.7% of respondents were uncertain about the availability of adequate programs for providing psychological support to students in the city. Additionally, 85.4% of students expressed the need to incorporate more mental health topics into the educational curriculum, and 86.9% advocated for the provision of free psychological support for youth [40]. Moreover, a meta-analysis and systematic review by Osborn et al. (2022) found that 21% (95% CI, 15–30%) of students globally sought psychological support, suggesting that students in Croatia are less likely to seek psychological help compared to students globally [20].

The systematic review had several limitations. Firstly, the study included only cross-sectional research, making it unjustifiable to draw conclusions about causality, meaning it is not possible to infer the direct impact of the COVID-19 pandemic on the mental health of students in Croatia based on these data. Additionally, this review encompassed studies that assessed student mental health using instruments such as the PHQ-9, GAD-7, and DASS-21. While these tools are effective for evaluating symptoms of depression, anxiety, and stress, they are not intended to provide formal diagnoses of mental disorders. Moreover, in the descriptive representation of the prevalence of symptoms of depression, anxiety, and stress, all participants who fell into one of the categories for these symptoms—mild, moderate, severe, and extremely severe—were included, which potentially contributes to an overestimation of the results. Additionally, Šimleša et al., Milić et al., and Romić et al. examined the mental health of students at the Zagreb School of Medicine, and although they were sampled during different periods, it is possible that a certain degree of overlap between study populations exists. However, we are unable to quantify or present this, particularly because the questionnaires were anonymous. Furthermore, based on the inclusion and exclusion criteria, this review included studies from three of the four major universities in Croatia (the University of Osijek, the University of Zagreb, and the University of Rijeka), with no studies from the University of Split.

## 5. Conclusions

The systematic review revealed a high prevalence of depression, anxiety, and stress symptoms among students in Croatia during the COVID-19 pandemic. This finding underscores the need for further longitudinal studies to explore the underlying risk factors associated with these mental health issues. Additionally, it highlights the importance of developing effective strategies for the early identification and management of mental disorders among students, regardless of the pandemic’s conclusion.

## Figures and Tables

**Table 1:** Inclusion and exclusion criteria of the study.

	Inclusion Criteria	Exclusion Criteria
Period of the study	Studies conducted during the COVID-19 pandemic (from 11 March 2020 to 5 May 2023)	Studies not conducted during the COVID-19 pandemic
Study design	Observational/cross-sectional study	Studies that are not observational/cross-sectional studies
Language	English, Croatian	Languages that are not English and Croatian
Participants	Student population	Non-student population
Study location (country)	Croatia	Countries other than Croatia
Study topic	Mental health (depression, anxiety, or stress) of students in Croatia during the COVID-19 pandemic	Studies that do not examine the mental health (depression, anxiety, or stress) of students in Croatia during the COVID-19 pandemic

**Table 2:** Methodological quality assessment of included studies using JBI Critical Appraisal Checklist for Studies Reporting Prevalence Data.

JBI Critical Appraisal Checklist for Studies Reporting Prevalence Data	Živić-Bećirević et al., 2020 [30]	Talapko et al., 2021 [31]	Romić et al., 2021 [32]	Šimleša et al., 2021 [33]	Milić et al., 2024 [34]
1. Was the sample frame appropriate to address the target population?	Yes	Yes	Yes	Yes	Yes
2. Were study participants sampled in an appropriate way?	Yes	Yes	Yes	Yes	Yes
3. Was the sample size adequate?	Yes	Unclear	Unclear	Unclear	Yes
4. Were the study subjects and the setting described in detail?	Yes	Yes	No	Yes	Yes
5. Was the data analysis conducted with sufficient coverage of the identified sample?	Not applicable	Not applicable	Not applicable	Not applicable	Not applicable
6. Were valid methods used for the identification of the condition?	Yes	Yes	Yes	Yes	Yes
7. Was the condition measured in a standard, reliable way for all participants?	Yes	Yes	Yes	No	Yes
8. Was there appropriate statistical analysis?	Yes	Yes	Yes	Yes	Yes
9. Was the response rate adequate, and if not, was the low response rate managed appropriately?	Unclear	Yes	Unclear	Unclear	Yes
The overall quality assessment score	77.8% (High)	77.8% (High)	55.6% (Medium)	55.6% (Medium)	90% (High)

**Table 3:** Key characteristics of the studies included in the systematic review.

Author	Study Design	Participants (*n*)	Male (*n*)	Female (*n*)	Period of the Study	Instrument
Živić-Bećirević et al., 2020 [30]	Cross-sectional study	923	674	249	May 2020.	DASS-21 *
Talapko et al., 2021 [31]	Cross-sectional study	823	153	670	May 2020–January 2021.	DASS-21 *
Romić et al., 2021 [32]	Cross-sectional study	280	146	102	January 2021.	PHQ-9 ^†^ GAD-7 ^‡^
Šimleša et al., 2021 [33].	Cross-sectional study	206	90	110	September 2021.	DASS-21 *
Milić et al., 2024 [34]	Cross-sectional study	2137	293	1844	March 2023.	PHQ-9 ^†^ GAD-7 ^‡^

* Depression, Anxiety, and Stress Scale-21, ^†^ 9-question Patient Health Questionnaire, ^‡^ General Anxiety Disorder 7-item scale.

**Table 4:** Prevalence of depression, anxiety, and stress symptoms among students in Croatia during the COVID-19 pandemic.

Author	Participants (*n*)	Depression Symptoms	Anxiety Symptoms	Stress Symptoms	Instrument
Živić-Bećirević et al., 2020 [30]	923	51.6%	-	-	DASS-21 *
Talapko et al., 2021 [31]	823	51.8%	51.9%	49.1%	DASS-21 *
Romić et al., 2021 [32]	280	65.2%	75.3%	-	PHQ-9 ^†^ GAD-7 ^‡^
Šimleša et al., 2021 [33]	206	25.7%	26.7%	15%	DASS-21 *
Milić et al., 2024 [34]	2137	76.5%	51.2%	-	PHQ-9 ^†^ GAD-7 ^‡^

* Depression, Anxiety, and Stress Scale-21, ^†^ 9-question Patient Health Questionnaire, ^‡^ General Anxiety Disorder 7-item scale.

## Data Availability

The data assessed and reported here can be obtained from the authors upon reasonable request and following ethical and privacy principles.
